# Divisive hierarchical maximum likelihood clustering

**DOI:** 10.1186/s12859-017-1965-5

**Published:** 2017-12-28

**Authors:** Alok Sharma, Yosvany López, Tatsuhiko Tsunoda

**Affiliations:** 1 Laboratory for Medical Science Mathematics, RIKEN Center for Integrative Medical Sciences, Yokohama, Kanagawa 230-0045 Japan; 2 Institute for Integrated and Intelligent Systems, Griffith University, Brisbane, QLD 4111 Australia; 3 School of Engineering & Physics, University of the South Pacific, Suva, Fiji; 4 Department of Medical Science Mathematics, Medical Research Institute, Tokyo Medical and Dental University, Tokyo, 113-8510 Japan; 5 CREST, JST, Tokyo, 113-8510 Japan

**Keywords:** Divisive approach, Hierarchical clustering, Maximum likelihood

## Abstract

**Background:**

Biological data comprises various topologies or a mixture of forms, which makes its analysis extremely complicated. With this data increasing in a daily basis, the design and development of efficient and accurate statistical methods has become absolutely necessary. Specific analyses, such as those related to genome-wide association studies and multi-omics information, are often aimed at clustering sub-conditions of cancers and other diseases. Hierarchical clustering methods, which can be categorized into agglomerative and divisive, have been widely used in such situations. However, unlike agglomerative methods divisive clustering approaches have consistently proved to be computationally expensive.

**Results:**

The proposed clustering algorithm (DRAGON) was verified on mutation and microarray data, and was gauged against standard clustering methods in the literature. Its validation included synthetic and significant biological data. When validated on mixed-lineage leukemia data, DRAGON achieved the highest clustering accuracy with data of four different dimensions. Consequently, DRAGON outperformed previous methods with 3-,4- and 5-dimensional acute leukemia data. When tested on mutation data, DRAGON achieved the best performance with 2-dimensional information.

**Conclusions:**

This work proposes a computationally efficient divisive hierarchical clustering method, which can compete equally with agglomerative approaches. The proposed method turned out to correctly cluster data with distinct topologies. A MATLAB implementation can be extraced from http://www.riken.jp/en/research/labs/ims/med_sci_math/ or http://www.alok-ai-lab.com

**Electronic supplementary material:**

The online version of this article (doi:10.1186/s12859-017-1965-5) contains supplementary material, which is available to authorized users.

## Background

In unsupervised clustering algorithms, the class label or the state of nature of a sample is unknown. The partitioning of data is then driven by considering similarity or distance measures. In some applications (e.g. genome-wide association studies, multi-omics data analyses), the number of clusters also remains unknown. Because such biological information usually tends to follow a normal distribution, the distribution of samples of each cluster can be assumed to be Gaussian.

Hierarchical clustering methods, which can be mainly categorized into agglomerative (bottom-up) and divisive (top-down) procedures, are well known [1–20]. In agglomerative procedures, each sample is initially assumed to be a cluster. The two nearest clusters (based on a distance measure or criterion function) are then merged at a time. This merger continues until all the samples are clustered into one group. Consequently, a tree like structure known as dendrogram is yielded. If the number of clusters is provided, the process of amalgamation of clusters can be terminated when the desired number of clusters is obtained. The first step of an agglomerative procedure considers all the possible mergers of two samples, which requires *n*(*n* − 1)/2 combinations (where *n* depicts the number of samples). Divisive procedures, on the other hand, perform clustering in an inverse way as compared to their agglomerative counterparts. They begin by considering a group (having all the samples) and divide it into two groups at each stage until all the groups comprise of only a single sample [21, 22]. In the first step of a divisive procedure all the partitions of a sample set are considered, which amounts to 2^*n* − 1^ − 1 combinations. This number of combinations grows exponentially and practically makes divisive clustering a difficult procedure to implement. However, there are a few divisive approaches which do not necessarily consider all the divisions [21]. In hierarchical classifications, each subcluster can be formed from one larger cluster split into two, or the union of two smaller clusters. In either case, false decisions made in early stages cannot be corrected later on. For this reason, divisive procedures, which start with the entire dataset, are in general considered safer than agglomerative approaches [21, 23]. Therefore, the accuracy of a divisive procedure is envisaged to be higher than that of an agglomerative procedure [24]. However, the high computational demand (*O*(2^*n*^)~*O*(*n* ^5^)) of divisive procedures has severely restricted their usage [24, 25] (though for special cases the complexity can be further reduced [26]). Therefore, the divisive procedure has not been generally used for hierarchical clustering, remaining largely ignored in the literature.

Hierarchical approaches do not require initial parameter settings and generally employed either linear or non-linear regression models [27–29]. Over the last few decades, a number of hierarchical approaches have been proposed. Some of these popular schemes are summarized below. The single linkage or link agglomerative hierarchical approach (SLink) [30] merges two adjacent neighbour groups. Euclidean distance for computing the proximity between two clusters. SLink is very sensitive to data location and occasionally generates groups in a long chain (called as chaining effect). This chaining effect can be reduced developing a method based on farthest distance. This was achieved by the complete linkage (CLink) hierarchical approach [2]. Nevertheless, CLink is also sensitive to outliers. Sensitiveness could be further decreased by the average linkage (ALink) hierarchical approach [31, 32]. ALink implements linking by using the average distance between two groups. In a similar way, the median linkage (MLink) hierarchical approach [33] regards median distance for linking. In Ward’s linkage (Wa-Link), clusters are merged based on the optimal value of an objective function [34]. In weighted average distance linkage (Wt-Link) hierarchical clustering [35, 36], the group sizes are not considered when computing average distances. Consequently, smaller groups will be assigned larger weights during the clustering process [35]. Similarly, model-based hierarchical clustering [4, 8] uses an objective function. Whereas the method in [8] follows a Bayesian analysis and uses both Dirichlet priors and multinomial likelihood function, the approach in [4] optimizes the distance between two GMMs. The number of group is previously defined. Most of these approaches are constructed using the agglomerative procedure, though their construction (with higher computational demand) is equally possible using the divisive procedure. Although divisive clustering is generally disregarded, some approaches like DIANA (DIvisive ANAlysis) program has been recently established [21]. In spite of well-established methods (i.e. EM algorithm [37, 38]) for estimating the parameters of a Gaussian mixture model, it is worth noting that hierarchical and expectation-maximization (EM) algorithms are very different in nature. The EM algorithm is an iterative optimization method, which requires prior knowledge of the number of clusters. It begins with a random choice of cluster centers and therefore returns different sets of clusters for distinct runs of the algorithm. Hierarchical clustering algorithms, on the other hand, do not require such prior knowledge and return a unique set of clusters. These advantages often make hierarchical clustering methods preferable to the EM algorithm for dealing with biological datasets where unique solutions are of utmost importance [39–41].

In this work, we described a new Divisive hieRArchical maximum likelihOod clusteriNg approach, abbreviated as DRAGON hereafter. This is a top-down procedure which does not find pairs. Instead, it takes out one sample at a time, maximally increasing the likelihood function. This process continues until the first cluster is obtained. This cluster is not further subdivided but removed from the sample set. In the remaining sample set, the same procedure is repeated for obtaining all the possible clusters. The removal of one sample out of *n* samples requires *n* search. This reduces the total search complexity to *O*(*n* ^2^ *c*) (where *c* is the number of clusters), which represents a significant reduction of the top-down procedure. The following sections present the mathematical derivation of the proposed model, and the analyses carried over on synthetic as well as on biological data to illustrate its usefulness.

## Methods

### Maximum likelihood clustering: an overview

This section summarizes an overview of the maximum likelihood method for clustering [22]. Here we are not introducing our method, instead we are proving a brief description of conventional maximum likelihood approach for clustering applications. It is possible to learn from an unlabeled data if some assumptions are taken. We will begin the section with an assumption that probability densities are known and it is required to estimate unknown parametric vector **θ**. The solution comes out to be similar to supervised learning case of maximum likelihood estimation. However, in the supervised learning case, the topology of groups of data is known. But in an unsupervised learning case one has to assume parametric form of data to reach to the solution. Here we describe how to estimate of maximum likelihood of clusters of a given sample set *χ*. The label of cluster of the sample sets is defined as *ω*. Assuming there are *c* clusters in the sample set (*c* ≥ 1), we define *Ω* = {*ω*_*j*_} (for *j* = 1, 2, …, *c*) as the cluster label for *j*th cluster *χ*_*j*_ (In many clustering problems, the number of *c* is unknown, this issue we will deal in detail in later section and in Additional file 1). In this paper, we followed the notations from Duda et al. [22] for the convenience of readers. Let a sample set *χ* = {**x**_1_, **x**_2_, …, **x**_*n*_} be defined in a *d*-dimensional space (It is assumed that *d* < *n*. For *d* ≫ *n*, dimensionality reduction techniques can be first applied for supervised or unsupervised learning tasks [42–47]). Let an unknown parameter vector be **θ** consisting of mean **μ** as well as covariance *Σ*. This will specify the mixture density as

1 $$ \mathrm{p}\left(\left.{\mathbf{x}}_{\mathrm{k}}\right|\boldsymbol{\uptheta} \right)={\sum}_{\mathrm{j}=1}^{\mathrm{c}}\mathrm{p}\left(\left.{\mathbf{x}}_{\mathrm{k}}\right|{\upomega}_{\mathrm{j}},{\boldsymbol{\uptheta}}_{\mathrm{j}}\right)\mathrm{P}\left({\upomega}_{\mathrm{j}}\right) $$

where *p*(**x**_*k*_|*ω*_*j*_, **θ**_*j*_) (for *j* = 1, …, *c*) is the conditional density, *P*(*ω*_*j*_) is the a priori probability and **θ** = {**θ**_*j*_}. The joint density is further defined using the log likelihood as

2 $$ L=\log p\left(\left.\chi \right|\boldsymbol{\uptheta} \right)=\log {\prod}_{k=1}^np\left(\left.{\mathbf{x}}_k\right|\boldsymbol{\uptheta} \right)={\sum}_{k=1}^n\log p\left(\left.{\mathbf{x}}_k\right|\boldsymbol{\uptheta} \right) $$

Assuming the joint density *p*(*χ*|**θ**) is differentiable w.r.t to **θ** then from Eqs. (1) to (2)

3 $$ {\nabla}_{{\boldsymbol{\uptheta}}_i}L={\sum}_{k=1}^n\frac{1}{p\left(\left.{\mathbf{x}}_k\right|\boldsymbol{\uptheta} \right)}{\nabla}_{{\boldsymbol{\uptheta}}_{\boldsymbol{i}}}\left[{\sum}_{j=1}^cp\left(\left.{\mathbf{x}}_k\right|{\omega}_j,{\boldsymbol{\uptheta}}_j\right)P\left({\omega}_j\right)\right] $$

where ${\nabla}_{{\boldsymbol{\uptheta}}_i}L$ is the gradient of *L* w.r.t. **θ**_*i*_. Assuming **θ**_*i*_ and **θ**_*j*_ are independent, and supposing a posteriori probability is

4 $$ P\left(\left.{\omega}_i\right|{\mathbf{x}}_k,\boldsymbol{\uptheta} \right)=\frac{p\left(\left.{\mathbf{x}}_k\right|{\omega}_i,{\boldsymbol{\uptheta}}_i\right)P\left({\omega}_i\right)}{p\left(\left.{\mathbf{x}}_k\right|\boldsymbol{\uptheta} \right)} $$

then from Eq. (4) we can observe that $\frac{1}{p\left(\left.{\mathbf{x}}_k\right|\boldsymbol{\uptheta} \right)}=\frac{P\left(\left.{\omega}_i\right|{\mathbf{x}}_k,\boldsymbol{\uptheta} \right)}{p\left(\left.{\mathbf{x}}_k\right|{\omega}_i,{\boldsymbol{\uptheta}}_i\right)P\left({\omega}_i\right)}$. Substituting this value in Eq. (3), we obtain

5 $$ {\nabla}_{{\boldsymbol{\uptheta}}_i}L={\sum}_{k=1}^nP\left(\left.{\omega}_i\right|{\mathbf{x}}_k,\boldsymbol{\uptheta} \right){\nabla}_{{\boldsymbol{\uptheta}}_i}\log p\left(\left.{\mathbf{x}}_k\right|{\omega}_i,{\boldsymbol{\uptheta}}_i\right) $$

Note that in Eq. (5), (1/*f*(*z*))*∇*_*z*_ *f*(*z*) is arranged as *∇*_*z*_ log *f*(*z*). Equation (5) can be equated to 0 (${\nabla}_{{\boldsymbol{\uptheta}}_i}L=0$) for obtaining maximum likelihood estimate ${\widehat{\boldsymbol{\uptheta}}}_i$. This will give us the solution as (interested readers may refer to Duda et al. [22] for further details.)

6 $$ P\left({\omega}_i\right)=\frac{1}{n}{\sum}_{k=1}^nP\left(\left.{\omega}_i\right|{\mathbf{x}}_k,\widehat{\boldsymbol{\uptheta}}\right) $$

7 $$ {\sum}_{k=1}^nP\left(\left.{\omega}_i\right|{\mathbf{x}}_k,\widehat{\boldsymbol{\uptheta}}\right){\nabla}_{{\boldsymbol{\uptheta}}_i}\log p\left(\left.{\mathbf{x}}_k\right|{\omega}_i,{\widehat{\boldsymbol{\uptheta}}}_i\right)=0 $$

8 $$ P\left(\left.{\omega}_i\right|{\mathbf{x}}_k,\widehat{\boldsymbol{\uptheta}}\right)=\frac{p\left(\left.{\mathbf{x}}_k\right|{\omega}_i,{\widehat{\boldsymbol{\uptheta}}}_i\right)P\left({\omega}_i\right)}{\sum_{j=1}^cp\left(\left.{\mathbf{x}}_k\right|{\omega}_j,{\widehat{\boldsymbol{\uptheta}}}_j\right)P\left({\omega}_j\right)} $$

In the case of normal distribution, the unknown mean and covariance {**μ**, *Σ*} parameters are replaced in **θ** in the Eqs. 6, 7 and 8 for yielding maximum likelihood estimates. The parameter **θ** is usually updated in an iterative fashion to attain $\widehat{\boldsymbol{\uptheta}}$ by EM algorithms or hill climbing schemes.

### DRAGON method: concept

**Fig. 1:**
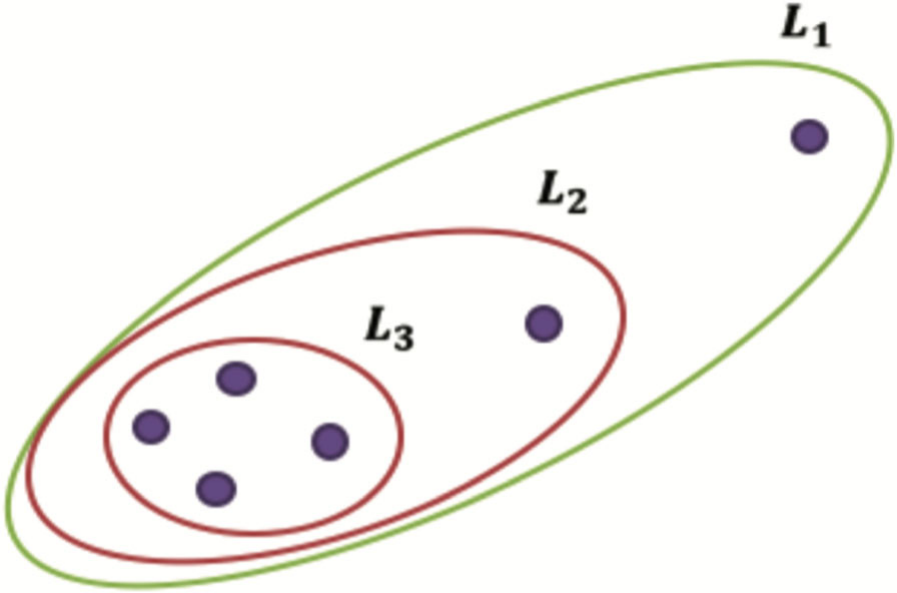
An illustration of the DRAGON method. This procedure results in the formation of one cluster. One sample is removed at a time, which maximally increases the likelihood function. At the beginning, the likelihood was *L*_1_ and after two iterations the likelihood became *L*_3_, where *L*_3_ > *L*_2_ > *L*_1_

Here we illustrate the clustering method DRAGON. In brief, the proposed procedure is top-down in nature. It initially considers the sample set as one cluster from which one sample is removed at a time. This increases the likelihood function and continues until the maximum likelihood is reached as depicted in Fig. 1 (where *L*_1_ is the cluster likelihood at the beginning of the process and *L*_3_ is the maximum likelihood after removing two samples). Once the first cluster is obtained it is removed from the sample set and the procedure is then repeated for attaining the subsequent clusters. Consequently, only one cluster will be retrieved from a sample set. It is assumed that samples are multinomial distributed, however, the number of clusters is not known at the beginning of the process.

To establish the maximum likelihood estimate in the divisive hierarchical context, we investigate the criterion function and the distance measure that satisfy it.

### DRAGON method: algorithm

To find the distance measure, we first define the log-likelihood function of a cluster *χ*_*s*_, where *χ*_*s*_ is a subset of *χ*. At the beginning, *χ*_*s*_ is the same as *χ*, however, in every subsequent iteration a sample $\widehat{\mathbf{x}}$ is removed from *χ*_*s*_ such that the likelihood function

9 $$ L={\sum}_{\mathbf{x}\in {\chi}_s}\log \left[p\left(\left.\mathbf{x}\right|\omega, \boldsymbol{\uptheta} \right)P\left(\omega \right)\right] $$

is maximized.

Since we are finding only one cluster in the sample set *χ*, a priori probability *P*(*ω*) can be ignored. We would like to explore how function *L* changes when a sample $\widehat{\mathbf{x}}$ is taken out. Let us suppose centroid **μ** and covariance *Σ* of *χ*_*s*_ are defined as

10 $$ \boldsymbol{\upmu} =\frac{1}{n}{\sum}_{\mathbf{x}\in {\chi}_s}\mathbf{x} $$

11 $$ \varSigma =\frac{1}{n}{\sum}_{\mathbf{x}\in {\chi}_s}\left(\mathbf{x}-\boldsymbol{\upmu} \right){\left(\mathbf{x}-\boldsymbol{\upmu} \right)}^{\mathrm{T}} $$

where the number of samples in *χ*_*s*_ is depicted as *n*. Assuming that the component density is normal then Eq. (9) can be simplified as

$$ {\displaystyle \begin{array}{c}L=\sum \limits_{\mathbf{x}\in {\chi}_s}\log \left[\frac{1}{{\left(2\pi \right)}^{d/2}{\left|\Sigma \right|}^{1/2}}\exp \left[-\frac{1}{2}{\left(\mathbf{x}-\boldsymbol{\upmu} \right)}^{\mathrm{T}}{\varSigma}^{-1}\left(\mathbf{x}-\boldsymbol{\upmu} \right)\right]\right]\\ {}=-\frac{1}{2} tr\left[{\varSigma}^{-1}{\sum}_{\mathbf{x}\in {\chi}_s}\left(\mathbf{x}-\boldsymbol{\upmu} \right){\left(\mathbf{x}-\boldsymbol{\upmu} \right)}^{\mathrm{T}}\right]-\frac{nd}{2}\log 2\pi -\frac{n}{2}\log \left|\varSigma \right|\end{array}} $$

where trace function is denoted by *tr*(). Since $tr\left[{\varSigma}^{-1}{\sum}_{\mathbf{x}\in {\chi}_s}\left(\mathbf{x}-\boldsymbol{\upmu} \right){\left(\mathbf{x}-\boldsymbol{\upmu} \right)}^{\mathrm{T}}\right]= tr\left({nI}_{d\times d}\right)= nd$, we can write *L* as

12 $$ L=-\frac{1}{2} nd-\frac{nd}{2}\log 2\pi -\frac{n}{2}\log \left|\varSigma \right| $$

If a sample $\widehat{\mathbf{x}}$ is removed from *χ*_*s*_ then centroid and covariance (Eqs. (10) and (11)) will change as follows

13 $$ {\boldsymbol{\upmu}}^{\ast }=\boldsymbol{\upmu} -\frac{\widehat{x}-\boldsymbol{\upmu}}{n-1} $$

14 $$ {\varSigma}^{\ast }=\frac{n}{n-1}\varSigma -\frac{n}{{\left(n-1\right)}^2}\left(\widehat{\mathbf{x}}-\boldsymbol{\upmu} \right){\left(\widehat{\mathbf{x}}-\boldsymbol{\upmu} \right)}^{\mathrm{T}} $$

In order to observe the alteration in the likelihood function (of Eq. (12)), we provide the following Lemma.

**Lemma 1** *Assume point* $\widehat{\boldsymbol{x}}$ *is taken out of a set χ*_*s*_ *and this changes the centroid and covariance (as Eqs. (* *13* *) and (* *14* *) described). Thereby the determinant of Σ* ^*^ *is defined as*

$$ \left|{\varSigma}^{\ast}\right|={\left(\frac{n}{n-1}\right)}^d\left|\varSigma \right|\left(1-\frac{1}{n-1}{\left(\widehat{\mathbf{x}}-\boldsymbol{\upmu} \right)}^{\mathrm{T}}{\varSigma}^{-1}\left(\widehat{\mathbf{x}}-\boldsymbol{\upmu} \right)\right) $$

*Proof* From Eq. (14), the determinant of *Σ* ^*^ will be

L1 $$ \left|{\varSigma}^{\ast}\right|=\left|\frac{n}{n-1}\varSigma -\frac{n}{{\left(n-1\right)}^2}\left(\widehat{\mathbf{x}}-\boldsymbol{\upmu} \right){\left(\widehat{\mathbf{x}}-\boldsymbol{\upmu} \right)}^{\mathrm{T}}\right| $$

For any square matrix of size *m* × *m*, we can write |*AB*| = |*A*||*B*|, and |*cA*| = *c* ^*m*^|*A*| where *c* is any scalar, This would enable us to write Eq. (L1) in the following manner

L2 $$ \left|{\varSigma}^{\ast}\right|={\left(\frac{n}{n-1}\right)}^d\left|\varSigma \right|\left|{I}_{d\times d}-\frac{1}{n-1}\left(\widehat{\mathbf{x}}-\boldsymbol{\upmu} \right){\left(\widehat{\mathbf{x}}-\boldsymbol{\upmu} \right)}^{\mathrm{T}}{\varSigma}^{-1}\right| $$

$\left|{I}_{m\times m}+ AB\right|$ can be proved to be |*I*_*n* × *n*_ + *BA*| by Sylvester’s determinant theorem (where *A* ∈ ℝ^*m* × *n*^ and *B* ∈ ℝ^*n* × *m*^ are rectangular matrices). This would allow us to write

$$ \left|{I}_{d\times d}-\frac{1}{n-1}\left(\widehat{\mathbf{x}}-\boldsymbol{\upmu} \right){\left(\widehat{\mathbf{x}}-\boldsymbol{\upmu} \right)}^{\mathrm{T}}{\varSigma}^{-1}\right|=\left|1-\frac{1}{n-1}{\left(\widehat{\mathbf{x}}-\boldsymbol{\upmu} \right)}^{\mathrm{T}}{\varSigma}^{-1}\left(\widehat{\mathbf{x}}-\boldsymbol{\upmu} \right)\right| $$

For any scalar |*c*| = *c*, the Lemma is then proved by substituting this term in Eq. (L1). □

It is now possible to define the change in *L* as

15 $$ {L}^{\ast }=L-\varDelta L $$

where *ΔL* is defined as

16 $$ \varDelta L=-\frac{1}{2}\log \left|\varSigma \right|+\frac{n-1}{2}\log \left(1-\frac{P}{n-1}\right)+\frac{n-1}{2}d\log \frac{n}{n-1}-\frac{d}{2}-\frac{d}{2}\log 2\pi $$

and *P* is expressed as

17 $$ P={\left(\widehat{\mathbf{x}}-\boldsymbol{\upmu} \right)}^{\mathrm{T}}{\varSigma}^{-1}\left(\widehat{\mathbf{x}}-\boldsymbol{\upmu} \right) $$

It can be observed from Eqs. (15) to (17) that when a sample $\widehat{\mathbf{x}}$ is taken out of cluster *χ*_*s*_, the change in *L* mainly depends on the term *P* as all the other terms are not changing. If we want to select $\widehat{\mathbf{x}}$ such that *L* ^*^ > *L*, this requires to solve the following maximization problem

18 $$ \widehat{\mathbf{x}}=\arg {\max}_{\mathbf{x}\in {\upchi}_{\mathrm{s}}}P $$

Therefore, by removing $\widehat{\mathbf{x}}$ the likelihood should increase until the maximum value is reached.

This procedure can track the location of the cluster having the highest density or likelihood. Because one sample is taken out at a time, it could be sensitive to data positions around the center of the cluster. Thereby, it is possible to locate the center of the cluster whereas its complete topology can be missed. In order to reduce such sensitiveness additional processing for tuning the cluster would be useful.

By taking out one sample at a time, we can obtain a cluster *χ*_*s*_ that provides maximum likelihood. All the samples taken out can be collated in a set defined as ${\chi}_s^{\ast }$, where ${\chi}_s\cup {\chi}_s^{\ast }=\chi$. The centroid of cluster *χ*_*s*_ can be obtained by **μ**_*s*_ = *E*[*χ*_*s*_]. It can be then employed to compute the distance of all the samples from **μ**_*s*_; i.e. *d*_*x*_ = *δ*(**x**, **μ**_*s*_) ∀ **x** ∈ *χ*, where *δ* denotes a distance measure (in this case the Euclidean metric) and *d*_*x*_ is a 1-dimensional sample or point corresponding to **x**. Thereafter, a centroid-based clustering scheme can be applied on this distance metric or inner product space (by considering 1-dimensional data and partitioning it into 2 groups) to readjust the cluster *χ*_*s*_. Here clustering is applied on a distance metric, which can be either the Euclidean norm or any form of kernel (as it is derived from dot product). This procedure can be repeated if **μ**_*s*_ is changing dramatically. The overall method is summarized in Table 1 and illustrated in Additional file 1 (slides 1–6).

**Table 1:** DRAGON Method

1. Given a sample set *χ*_*s*_ (at the beginning *χ*_*s*_ = *χ*), compute likelihood *L* (Eq. (12)). 2. Until *L* ^*^ > *L*, remove one sample $\widehat{\mathbf{x}}\in {\chi}_s$ (Eq. (18)), compute new likelihood *L* ^*^, update *χ*_*s*_ and *L*. 3. Find centroid **μ**_*s*_ = *E*[*χ*_*s*_] and *d*_*x*_ = *δ*(**x**, **μ**_*s*_) ∀ **x** ∈ *χ*. 4. Partition {*d*_*x*_} into two groups, for example using k-means algorithm (or divide into two groups based on their values). One of these groups will have lower *d*_*x*_ values (representing closeness to **μ**_*s*_) whereas the other will have higher *d*_*x*_ values (representing distance from **μ**_*s*_). Update *χ*_*s*_ by replacing it with the samples with the lower *d*_*x*_ values. 5. If required repeat steps 3 and 4. Take out the cluster *χ*_*s*_ from *χ*. Update *χ* accordingly (the updated *χ* would contain all the samples except *χ*_*s*_; i.e. *χ* ∩ *χ*_*s*_ = *Φ*). 6. Repeat all the steps until all the possible clusters (or desired number of clusters) are obtained.	

The next issue is the estimation of the number of clusters (*c*). If the value of *c* were given, it is then easier to find the locations. However, in some applications, *c* is unknown. In such situations, a range of values of *c* can be inserted to the procedure so that the best value in the range can be estimated. If no clue about *c* were given, the maximum number of possible clusters *C* can be investigated and the best among them (*c* ≤ *C*) can be chosen. In order to estimate *c*, we first define the total likelihood function as

19 $$ {L}_t(c)={\sum}_{i=1}^c{L}_i $$

where *L*_*i*_ is the likelihood of *i*th cluster. The total likelihood function *L*_*t*_ can be computed for different values of *c*. If for a particular number of clusters (*k*), the variation between two successive total likelihood functions were not significant, then we can estimate *c* to be *k*. Let the difference between two successive total likelihoods be *δL*_*t*_(*k*) = *L*_*t*_(*k* + 1) − *L*_*t*_(*k*), this quantity can be normalized as

20 $$ \delta {L}_t\leftarrow \frac{\delta {L}_t-\min \left(\delta {L}_t\right)}{\max \left(\delta {L}_t\right)-\min \left(\delta {L}_t\right)} $$

where *δL*_*t*_ is normalized over all the possible values of *k*.

Figure S5 in Additional file 1 illustrates the above explanation with a dataset of 4 clusters.

### DRAGON method: search complexity

In this section, we briefly discuss the search complexity of the DRAGON method. As explained above the proposed method begins by taking one sample out of the sample set, which increases the likelihood function and requires *n* search. However, in the second iteration the search reduces to *n* − 1. Finding a cluster having *n*_1_ samples requires (1/2)(*n* − *n*_1_)(*n* + *n*_1_ + 1) total search (see Additional file 2 for details). Therefore, the search for *c* clusters results *O*(*n* ^2^ *c*). It should be noted here that this search in conventional divisive hierarchical approaches is quite expensive, in the order of *O*(2^*n*^). However, the search of DRAGON is in the order of *O*(*n* ^2^ *c*), which indicates a considerable reduction. Furthermore, DRAGON employs the k-means clustering algorithm in the dot product space as an intermediate step. The computational complexity of k-means is considered to be linear (e.g. using Lloyd’s algorithm this is *O*(2*nt*) because dimensionality is 1 in an intermediate step, and the number of classes is 2. Here, *t* represents the number of iterations).

## Results and discussion

To validate the DRAGON method, we performed analyses using synthetic and biological data. We further compared its clustering accuracy with that of existing hierarchical methods.

### Analysis on synthetic data

**Fig. 2:**
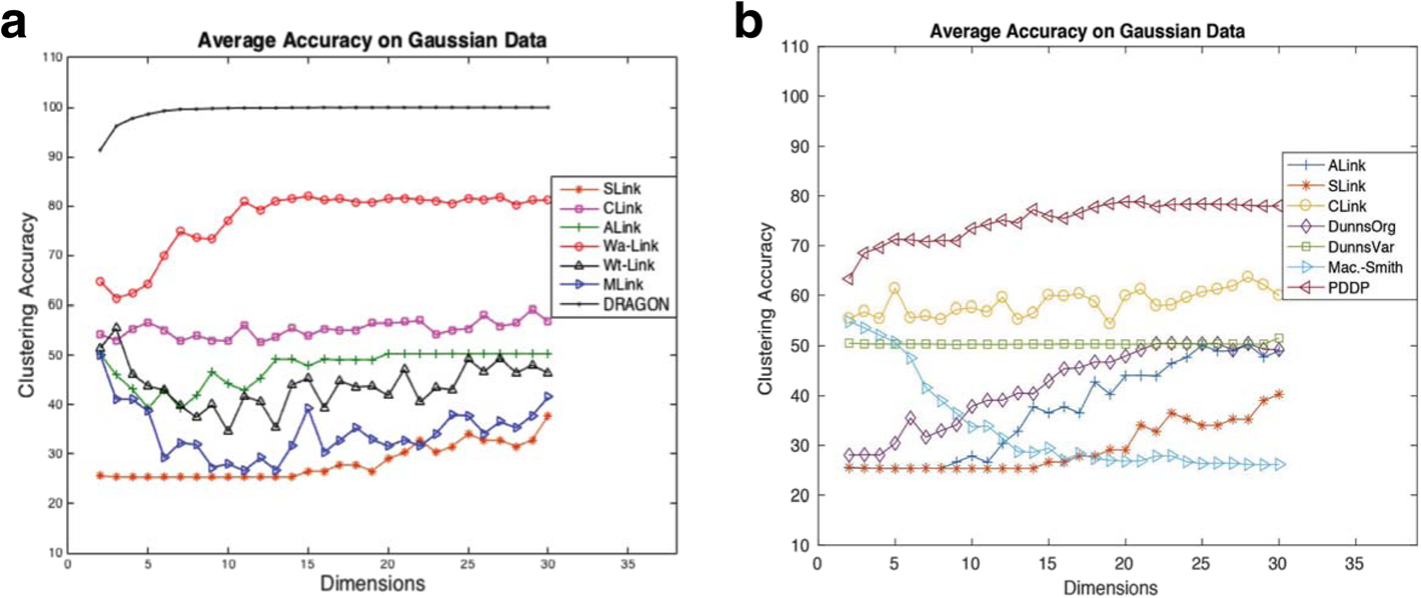
Average clustering accuracy (over 20 attempts) on synthetic data with four clusters for (**a**) DRAGON and various agglomerative hierarchical methods, and (**b**) divisive hierarchical methods

For the analysis on synthetic data, we generated Gaussian data of dimensionality *d* with 4 clusters. This data consisted of 400 samples with similar topology to that described in Figure S1 of Additional file 1). With the help of different random seeds we produced the data 20 times, and for on each occasion we calculated the clustering accuracy. We then computed the average or mean of clustering accuracy over 20 attempts to have a statistically stable value. The dimension of the generated data was increased from 2 to 30. For evaluation purposes, we also used other agglomerative hierarchical methods such as SLink, CLink, MLink, ALink, Wa-Link, and Wt-Link. The average clustering accuracies of these approaches over dimensionality *d* are shown in Fig. 2a. For all the above methods, we provided the number of clusters; i.e. *c* = 4. For the DRAGON method (of Table 1), we iterated two times (step 5 of Table 1) in all the experiments. Additionally, we assessed seven previously compared divisive hierarchical methods [24]: ALink (average link), SLink (single link), CLink (complete link), DunnsOrg (Dunn’s original), DunnsVar (Dunn’s variant), Mac-Smith (Macnaughton-Smith) and PDDP (Principal Direction). The average clustering accuracies of these divisive methods are summarized in Fig. 2b. As Fig. 2b shows when the dimensionality of data increases, Mac-Smith performs poorly whereas ALink, SLink and DunnsOrg slightly improve their performances. The data dimension did not affect the accuracy of DunnsVar whatsoever, which remains around 50%. The highest accuracy is achieved by the PDDP method (roughly 80%), however, this accuracy is still lower than that of DRAGON.

It can be observed from Fig. 2 that on Gaussian data, the DRAGON method provides promising results over other hierarchical methods either agglomerative or divisive. The Wa-Link hierarchical method also shows good results after the DRAGON method over dimensionality *d* = 2, …, 30.

To avoid limiting our validation to Gaussian datasets, we carried out additional analyses on synthetic data that included Pathbased [48], Flame [49] and Aggregation [50] datasets. The results are summarized in Tables S1.1, S1.2 and S1.3 of Additional file 1.

### Analysis on biological data

We also utilized biological datasets, namely acute leukemia [51], mixed-lineage leukemia (MLL) [52] and mutation data from The Cancer Genome Atlas for assessing clustering accuracy of several hierarchical approaches studied in this paper. A summary of datasets can be found below:

Acute leukemia dataset –comprises DNA microarray gene expressions. The samples belong to acute leukemias of humans. Two kinds are available: acute myeloid leukemia (AML) and acute lymphoblastic leukemia (ALL). The dataset has 25 AML and 47 ALL bone marrow samples with a dimension of 7129.

MLL dataset –has three ALL, MLL and AML classes. It contains 72 leukemia samples where 24 belong to ALL, 20 belong to MLL and 28 belong to AML with dimensionality 12,582.

Mutation dataset – this dataset is derived from The Cancer Genome Atlas project (https://tcga-data.nci.nih.gov/docs/publications/tcga/?). It includes mutation data for breast cancer, glioblastoma, kidney cancer and ovarian cancer. The data is divided into two groups of 416 samples and 90 samples, which contain 1636 genes.

To vary its number of features or dimensions, we employed the Chi-squared method for ranking the genes (the InfoGain feature selection method was also employed, see Additional file 3). We then performed clustering on samples and computed the clustering accuracy on dimensionality *d* = 2, …, 5. The clustering accuracies (was measured with the package AccMeasure 2011: http://www.mathworks.com/matlabcentral/fileexchange/32197-clustering-results-measurement/content/AccMeasure.m) on acute leukemia, MLL and mutation datasets are depicted in Tables 2, 3 and 4, respectively. The best outcomes are highlighted in bold faces.

**Table 2:** Clustering accuracy (%) on acute leukemia dataset

Methods	Dim 2	Dim 3	Dim 4	Dim 5
SLINK	66.7	66.7	66.7	66.7
CLINK	84.7	81.9	81.9	81.9
ALINK	76.4	81.9	84.7	84.7
Wa-LINK	**94.4**	81.9	81.9	81.9
Wt-LINK	**94.4**	81.9	81.9	81.9
MLINK	**94.4**	81.9	81.9	81.9
SLINK (Divisive)	66.7	66.7	66.7	66.7
CLINK (Divisive)	80.6	80.6	80.6	80.6
ALINK (Divisive)	66.7	66.7	66.7	66.7
Dunn’s original (Divisive)	76.4	80.6	80.6	80.6
Dunn’s variant (Divisive)	72.2	70.8	70.8	72.2
Macnaughton-Smith (Divisive)	86.1	81.9	81.9	81.9
Principal Direction (Divisive)	89.4	88.9	88.9	88.9
K-means	90.3	89.5	81.9	81.9
DRAGON	93.1	**97.2**	**97.2**	**94.4**

**Table 3:** Clustering accuracy (%) on MLL dataset

Methods	Dim 2	Dim 3	Dim 4	Dim 5
SLINK	40.3	40.3	43.1	43.1
CLINK	45.8	50.0	54.2	72.2
ALINK	50.0	50.0	50.0	72.2
Wa-LINK	62.5	**62.5**	62.5	**84.7**
Wt-LINK	45.8	50.0	43.1	69.4
MLINK	45.8	50.0	43.1	69.4
SLINK (Divisive)	41.7	41.7	43.1	43.1
CLINK (Divisive)	54.2	45.8	56.9	72.2
ALINK (Divisive)	41.7	41.7	43.1	72.2
Dunn’s original (Divisive)	44.4	44.4	45.8	72.2
Dunn’s variant (Divisive)	41.7	41.7	43.1	73.6
Macnaughton-Smith (Divisive)	54.2	48.6	50.0	72.2
Principal Direction (Divisive)	62.5	**62.5**	62.5	81.9
K-means	56.0	57.0	58.1	61.6
DRAGON	**65.3**	**62.5**	**68.1**	**84.7**

**Table 4:** Clustering accuracy (%) on mutation dataset

Methods	Dim 2	Dim 3	Dim 4	Dim 5
SLINK	**77.9**	77.9	77.9	82.4
CLINK	77.3	82.0	**82.8**	**83.2**
ALINK	77.3	77.3	**82.8**	82.4
Wa-LINK	77.3	77.7	77.9	77.9
Wt-LINK	77.3	82.0	**82.8**	54.6
MLINK	54.6	54.6	54.6	82.4
SLINK (Divisive)	54.5	54.5	54.5	82.2
CLINK (Divisive)	54.4	54.5	77.3	77.3
ALINK (Divisive)	**77.9**	82.6	82.6	82.4
Dunn’s original (Divisive)	77.3	**83.0**	77.3	82.4
Dunn’s variant (Divisive)	54.5	54.5	54.5	77.3
Macnaughton-Smith (Divisive)	**77.9**	**83.0**	**82.8**	82.4
Principal Direction (Divisive)	54.5	54.5	54.5	54.5
K-means	64.9	67.1	65.7	63.3
DRAGON	**77.9**	82.2	82.2	82.2

It is noticed from Table 2 that Wa-Link, Wt-Link and MLink achieve the highest performance when *d* = 2. For the other dimensions (*d* = 3, 4 and 5), DRAGON shows the highest performance. From Table 3, it can be seen that DRAGON achieves reasonable performance for all the dimensions. On mutation data (Table 4), DRAGON is showing the highest performance for *d* = 2 and slightly low performance (82.2%) for the other dimensions. Dunn’s original and Macnaughton-Smith provide the highest performance when *d* = 3. Despite the reasonable performance of divisive clustering methods in Table 4, it is worth noting that their running times were extremely slow, specially when the number of samples increases as it is the case with mutation data. In general, it can be summarized that the DRAGON method exhibited promising results in terms of clustering accuracy over other hierarchical methods. Also its search complexity is *O*(*n* ^2^ *c*), which is significantly lower than that of conventional divisive approaches.

## Conclusions

In this work, we proposed a divisive hierarchical maximum likelihood clustering method whose search complexity was reduced to *O*(*n* ^2^ *c*). Its overall clustering accuracy showed a significant improvement over that of agglomerative hierarchical clustering methods when compared on both synthetic and biological datasets.

## Additional files

Additional file 1: Divisive hierarchical maximum likelihood clustering. In this file an illustration of DRAGON method is given. Additionally, performance (in terms of Rand index) is given for synthetic data (Flame, Pathbased and Aggregation). (PDF 963 kb)

Additional file 2: Computational consideration of DRAGON search. In this file derivation of computational complexity of DRAGON search is given. (PDF 84 kb)

Additional file 3: Clustering accuracy using InfoGain feature selection method. In this file, InfoGain filtering method was used to perform feature selection. Thereafter, various clustering methods were used to evaluate the performance of DRAGON method. (PDF 68 kb)

## Abbreviations

ALL: Acute lymphoblastic leukemia
AML: Acute myeloid leukemia
CLink: Complete linkage
DIANA: Divisive analysis
DRAGON: Divisive hierarchical maximum likelihood clustering
EM: Expectation-maximization
GMM: Gaussian mixture model
ML: Maximum likelihood
MLink: Median linkage
MLL: Mixed lineage leukemia
SLink: Single linkage
Wa-Link: Ward’s linkage
WLink: Weighted average distance linkage
Wt-Link: Weighted linkage
