# Supplementary material for: Divisive hierarchical maximum likelihood clustering
Source: BMC Bioinformatics. 2017 Dec 28;18(Suppl 16):546. doi: 10.1186/s12859-017-1965-5 (PMC5751574; doi:10.1186/s12859-017-1965-5)
Supplement: Supplementary file 1 — Divisive hierarchical maximum likelihood clustering. In this file an illustration of DRAGON method is given. Additionally, performance (in terms of Rand index) is given for synthetic data (Flame, Pathbased and Aggregation). (PDF 963 kb) [file 12859_2017_1965_MOESM1_ESM.pdf]

Supplement 1:

Divisive hierarchical maximum likelihood clustering

Illustration using 4 cluster case

Synthetic data topology

4 cluster case

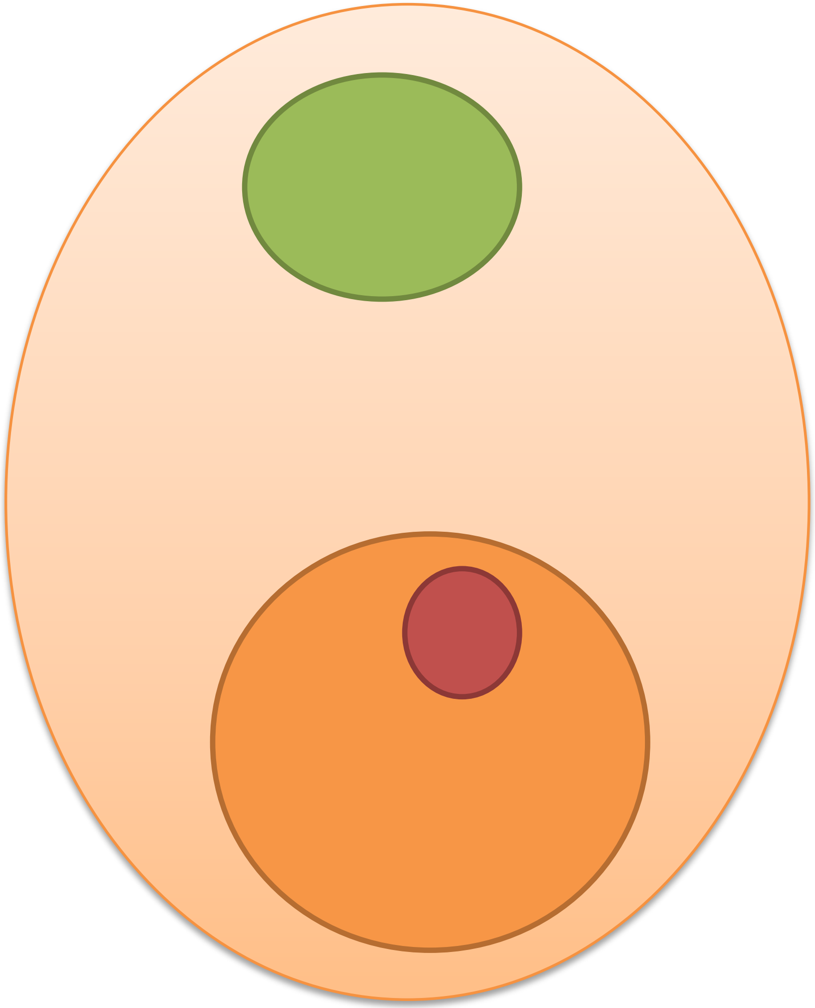

Fig. S1a

Description in 2D-plane

Determine location (mean μ)

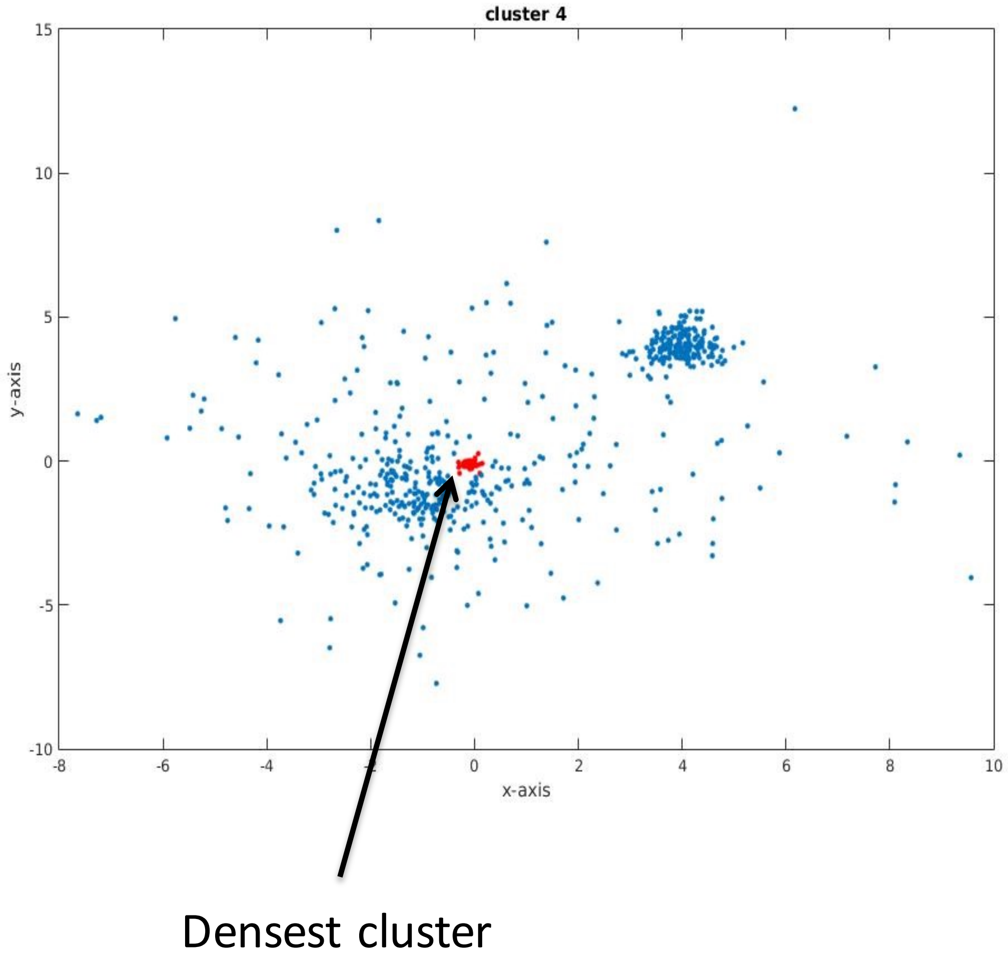

Fig. S1b

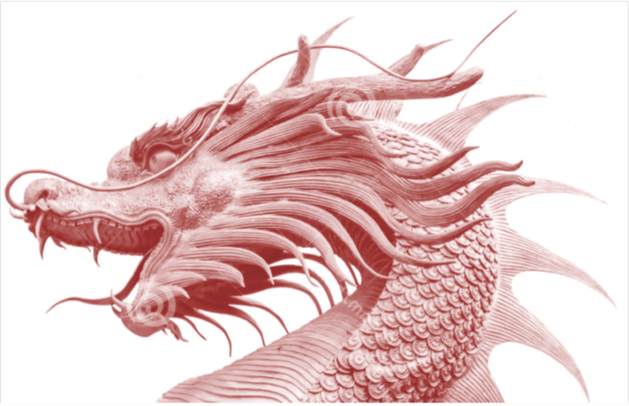

Dragon method: an illustration

- Two steps
  1. Finding location.
  2. Tuning cluster.

STEP 1: Finding Location

Take out only 1 sample at a time such that Likelihood is increased

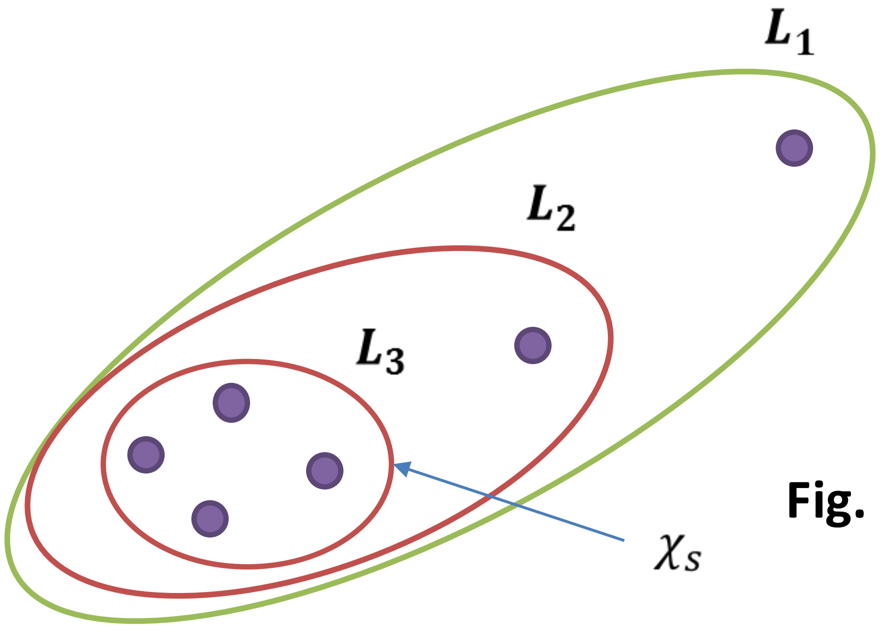

Fig. S2

Maximize distance P

$$\hat{x} = \arg\max_{x \in \chi} P \tag{1}$$

where $P = (x - \mu)^T \Sigma^{-1} (x - \mu)$ (2)

Remove $\hat{x} \in \chi$ such that $L^* > L$ (i.e., new L > old L)

This procedure gives a cluster χ_s

Step 2: Tuning cluster

- From the mean of χ_s, find distance (or dot product) to all the samples.
- Perform k-means in this inner product space.

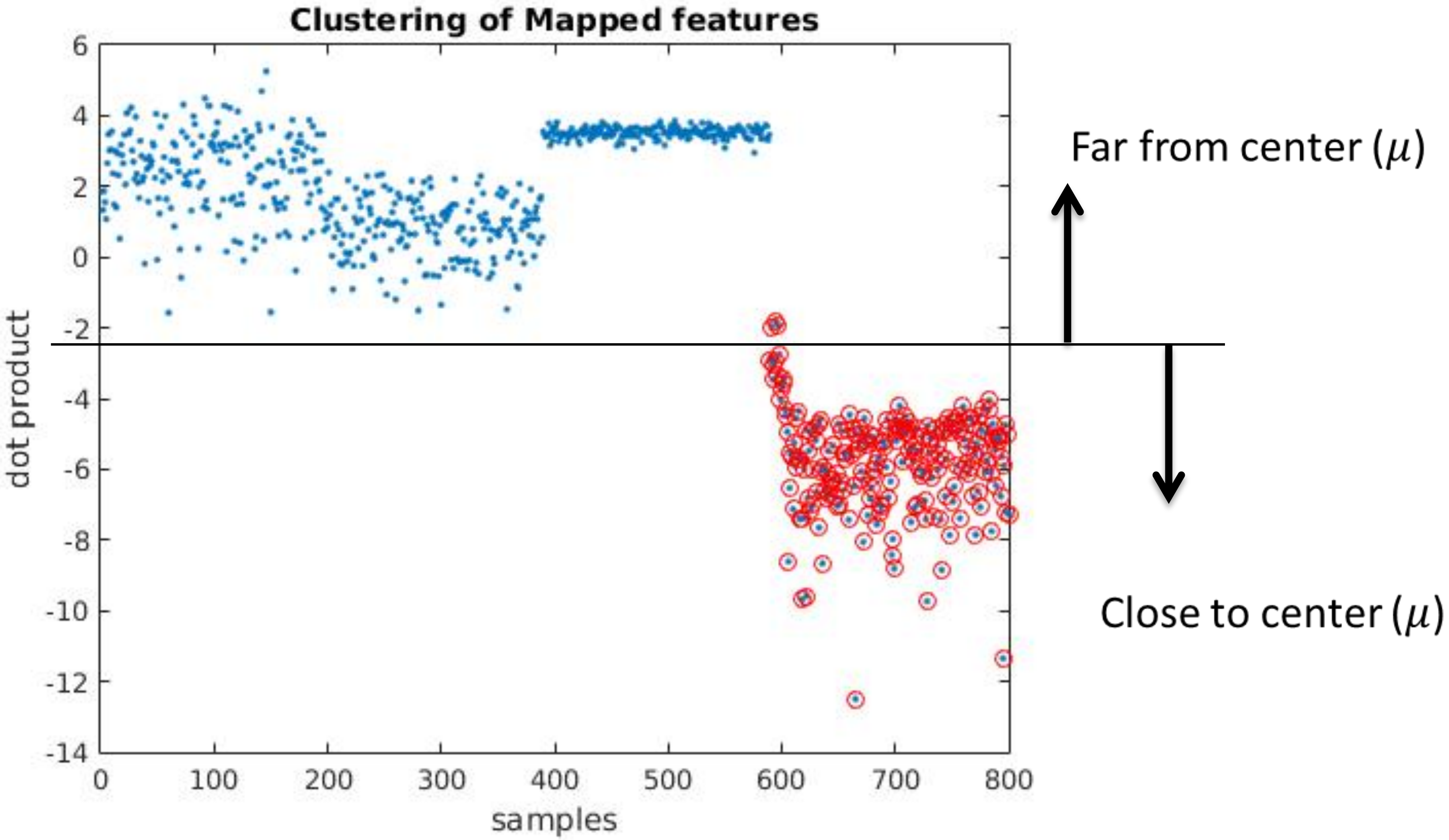

Fig. S3

- Conducting the same 2-step procedure to all the remaining clusters one-by-one (assuming 4-clusters), we get Fig. S4.

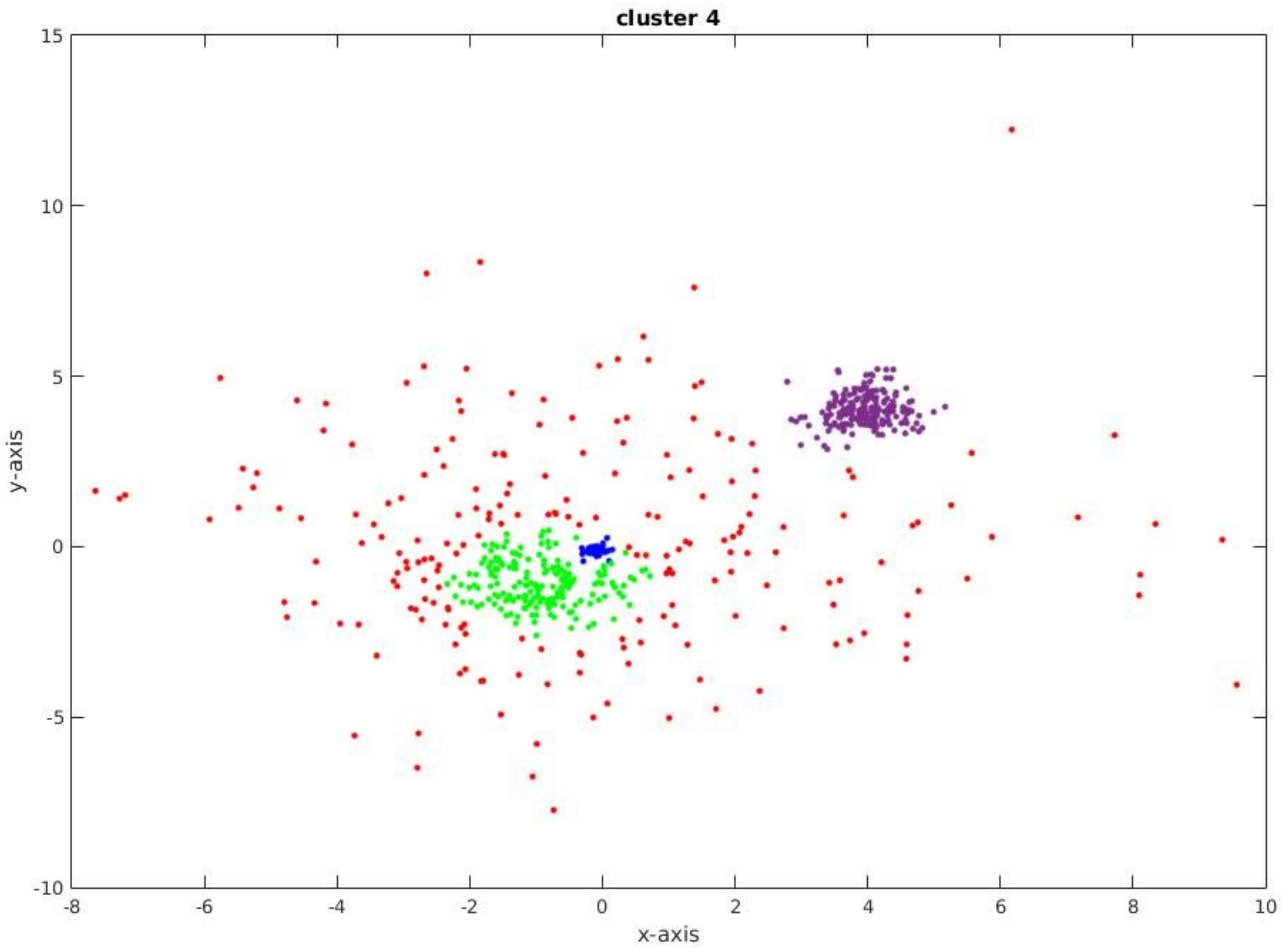

Fig. S4

Ltot and δLtot plots
(to identify number of clusters)

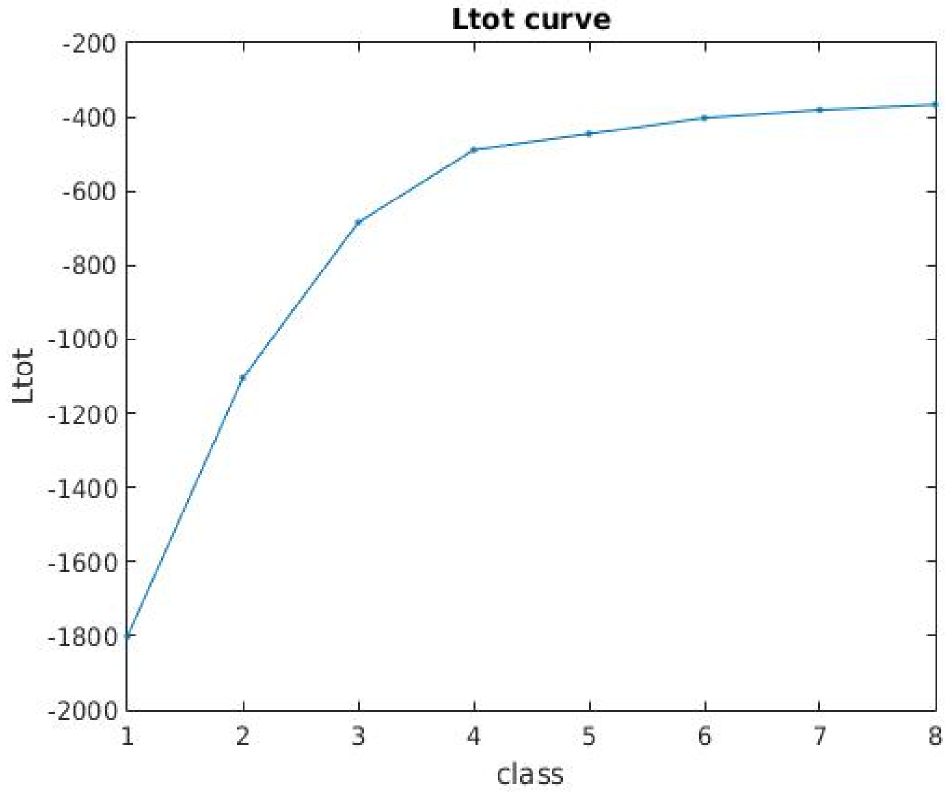

Fig. S5a

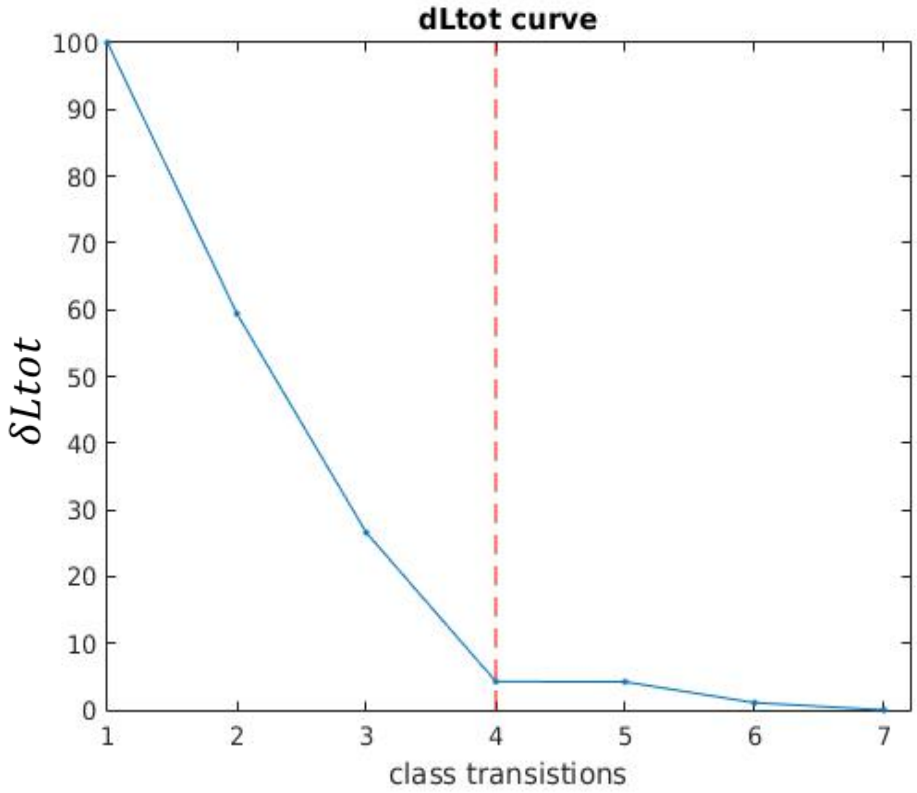

Fig. S5b

Computation of $\delta Ltot$ curve

$\delta Ltot \leftarrow diff(Ltot)$

$\delta Ltot \leftarrow \delta Ltot - \min(\delta Ltot)$

$\delta Ltot \leftarrow \frac{\delta Ltot}{\max(\delta Ltot)}$ (to have a ranged between [0,1] or [0,100] if mult. By 100)

Synthetic data: Flame

Flame data was provided by Fu and Medico, 2007

Table S1.1

Methods	Rand Score
SLINK	0.54
CLINK	0.50
ALINK	0.72
Wa-LINK	0.60
Wt-LINK	0.70
MLINK	0.68
SLINK (Div)	0.54
CLINK (Div)	0.50
ALINK (Div)	0.54
Dunn's original (Div)	0.54
Dunn's variant (Div)	0.50
Macnaughton-Smith et al. (Div)	0.51
Principal Direction (Div)	0.56
Kmeans	0.73
DRAGON	0.95

Flame: N=240, nClusters=2, nDimensions=2: L. Fu and E. Medico, FLAME, a novel fuzzy clustering method for the analysis of DNA microarray data. *BMC bioinformatics*, 2007. 8(1): p. 3.

Synthetic data: Pathbased

Pathbased data was provided by Chang and Yeung, 2008

Table S1.2

Methods	Rand Score
SLINK	0.34
CLINK	0.69
ALINK	0.74
Wa-LINK	0.76
Wt-LINK	0.71
MLINK	0.75
SLINK (Div)	0.34
CLINK (Div)	0.63
ALINK (Div)	0.73
Dunn's original (Div)	0.72
Dunn's variant (Div)	0.70
Macnaughton-Smith et al. (Div)	0.72
Principal Direction (Div)	0.68
Kmeans	0.75
DRAGON	0.93

Pathbased: N=300, nClusters=3, nDimensions=2: H. Chang and D.Y. Yeung, Robust path-based spectral clustering. *Pattern Recognition*, 2008. 41(1): p. 191-203.

Synthetic data: Aggregation

Aggregation data was provided by Gionis et al., 2007

Table S1.3

Methods	Rand Score
SLINK	0.93
CLINK	0.93
ALINK	1.00
Wa-LINK	0.94
Wt-LINK	0.92
MLINK	0.98
SLINK (Div)	0.75
CLINK (Div)	-
ALINK (Div)	0.72
Dunn's original (Div)	0.77
Dunn's variant (Div)	0.77
Macnaughton-Smith et al. (Div)	0.83
Principal Direction (Div)	0.84
Kmeans	0.92
DRAGON	0.98

Pathbased: N=788, nClusters=7, nDimensions=2: A. Gionis, H. Mannila, and P. Tsaparas, Clustering aggregation. *ACM Transactions on Knowledge Discovery from Data (TKDD)*, 2007. 1(1): p. 1-30.
