# Supplementary material for: Divisive hierarchical maximum likelihood clustering
Source: BMC Bioinformatics. 2017 Dec 28;18(Suppl 16):546. doi: 10.1186/s12859-017-1965-5 (PMC5751574; doi:10.1186/s12859-017-1965-5)
Supplement: Supplementary file 2 — Computational consideration of DRAGON search. In this file derivation of computational complexity of DRAGON search is given. (PDF 84 kb) [file 12859_2017_1965_MOESM2_ESM.pdf]

# Supplement 2

## Computational consideration of DRAGON search

Let $n$ be the total number of samples in a sample set $\chi$, and $n_i$ be the number of samples in $i$th cluster ($\chi_i$). By taking one sample out at a time would induce the following computational requirements for dragon search:

| $k$-th sample taken out | Remaining number of samples after $k$-th sample was taken out | Search requirements |
|---|---|---|
| 1st sample taken out | $n-1$ | $n$ |
| 2nd sample taken out | $n-2$ | $n-1$ |
| ⋮ | ⋮ | ⋮ |
| ⋮ | ⋮ | ⋮ |
| $n-n_1$ sample taken out | $n-(n-n_1)$ | $n-(n-n_1-1)$ |

The above search would find the first cluster with $n_1$ samples. It can be noted (from column 3 of the table) that $n$ is appeared $n-n_1$ times (along the row). Therefore, the total search to obtain the first cluster would be the summation of column 3; i.e.,

$$\begin{aligned} T_1 &= n(n-n_1) - \tfrac{1}{2}(n-n_1-1)(n-n_1) \\ &= \tfrac{1}{2}(n-n_1)(n+n_1+1) \\ &= \tfrac{1}{2}\left(n+\tfrac{1}{2}\right)^2 - \tfrac{1}{2}\left(n_1+\tfrac{1}{2}\right)^2 \end{aligned}$$

assuming $n+\frac{1}{2} \approx n$ and $n_1+\frac{1}{2} \approx n_1$, we get

$$T_1 \approx \tfrac{1}{2}n^2 - \tfrac{1}{2}n_1^2 \qquad \text{(S1)}$$

After locating cluster 1, $n_1$ samples of cluster 1 will be removed from the sample set $\chi$. This would reduce the total number of samples to $n-n_1$. Therefore, for the 2nd cluster the search can be obtained by simply replacing $n$ by $n-n_1$, and, $n_1$ by $n_2$ in Equation S1, where $n_2$ is the number of samples in cluster 2. This would give total search for the 2nd cluster as

$$T_2 \approx \tfrac{1}{2}(n-n_1)^2 - \tfrac{1}{2}n_2^2 \qquad \text{(S2)}$$

Similarly, the search for $k$-th cluster can be given obtained as

$$T_k \approx \tfrac{1}{2}(n-n_1-n_2 \ldots - n_{k-1})^2 - \tfrac{1}{2}n_k^2 \qquad \text{(S3)}$$

If there are $c$ clusters then the total search would be:

$$T_{tot} \approx \tfrac{1}{2}\textstyle\sum_{i=1}^{c-1}(n-n_1-n_2 \ldots n_{i-1})^2 - \tfrac{1}{2}\textstyle\sum_{i=1}^{c-1} n_i^2 \qquad \text{(S4)}$$

Note that in Equation S4, the upper limit of summation is $c-1$ and not $c$ because if there are only two clusters required to be found then only $T_1$ search is required. In other words, the 1st cluster ($\chi_1$) can be

obtained by removing a sample at a time from sample set $\chi$ (this would require $T_1$ search), and, the remaining samples can be collected to form the second cluster (this would require no search). Similarly, if there are $c$ clusters to be found then $T_1 + T_2 + \cdots + T_{c-1}$ search is required. Equation S4 can be simplified as

$$T_{tot} \approx \frac{1}{2}\sum_{i=1}^{c-1}\left(n - \sum_{j=1}^{i-1} n_j\right)^2 - \frac{1}{2}\sum_{i=1}^{c-1} n_i^2 \qquad \text{(S5)}$$

Two cases can be considered to further simply Equation S5: 1) If all clusters have equal number of samples; i.e., $n_1 = n_2 = \cdots = n_{c-1} = n_c = n/c$ ; and, 2) if the number of clusters is same as the number of samples ($n = c$); i.e., each cluster would have 1 sample each or $n_i = 1$.

<u>Case 1</u>: if $n_i = n/c$ for $i = 1,2, \dots, c$ then from Equation S5

$$T_{tot} \approx \frac{1}{2}\sum_{i=1}^{c-1}\left(n - \frac{n}{c}(i-1)\right)^2 - \frac{1}{2}\frac{n^2}{c^2}(c-1)$$
$$= \frac{1}{2}\frac{n^2}{c^2}\sum_{i=1}^{c-1}\left(c - (i-1)\right)^2 - \frac{1}{2}\frac{n^2}{c^2}(c-1)$$

substituting $i \leftarrow c - (i-1)$, we get

$$= \frac{1}{2}\frac{n^2}{c^2}\sum_{i=2}^{c} i^2 - \frac{1}{2}\frac{n^2}{c^2}(c-1)$$

from the sum of the squares of the first $c$ natural numbers we get

$$= \frac{1}{2}\frac{n^2}{c^2}\left(\frac{c^3}{3} + \frac{c^2}{2} + \frac{c}{6} - 1\right) - \frac{1}{2}\frac{n^2}{c^2}(c-1)$$

assuming $c - 1 \approx c$ and $c^3 \gg c^2 \gg c$, we get

$$T_{tot} \approx \frac{1}{6}n^2 c - \frac{1}{2}\frac{n^2}{c} = O(n^2 c)$$

<u>Case 2</u>: If $n_i = 1$ for $i = 1,2, \dots, n$ (as $n = c$) then from Equation S5

$$T_{tot} \approx \frac{1}{2}\sum_{i=1}^{n-1}\left(n - \sum_{j=1}^{i-1} n_j\right)^2 - \frac{1}{2}\sum_{i=1}^{n-1} n_i^2$$
$$= \frac{1}{2}\sum_{i=1}^{n-1}(n - (i-1))^2 - \frac{1}{2}(n-1)$$

substituting $i \leftarrow n - (i-1)$, we get

$$= \frac{1}{2}\sum_{i=2}^{n-1} i^2 - \frac{1}{2}(n-1)$$
$$= \frac{1}{2}\left(\frac{n^3}{3} + \frac{n^2}{2} + \frac{n}{6} - 1\right) - \frac{1}{2}(n-1)$$

assuming $n^3 \gg n^2 \gg n$, we get

$$T_{tot} \approx \frac{1}{6}n^3 = O(n^3)$$

Therefore, the search complexity is between $O(n^2 c)$ and $O(n^3)$.
