# Supplementary material for: Divisive hierarchical maximum likelihood clustering
Source: BMC Bioinformatics. 2017 Dec 28;18(Suppl 16):546. doi: 10.1186/s12859-017-1965-5 (PMC5751574; doi:10.1186/s12859-017-1965-5)
Supplement: Supplementary file 3 — Clustering accuracy using InfoGain feature selection method. In this file, InfoGain filtering method was used to perform feature selection. Thereafter, various clustering methods were used to evaluate the performance of DRAGON method. (PDF 68 kb) [file 12859_2017_1965_MOESM3_ESM.pdf]

Supplement 3

Clustering accuracy using InfoGain feature selection method

This supplement reports the clustering accuracy of various methods on ALL, MLL and Mutation datasets. The genes from the datasets were pre-selected using InfoGain ranking scheme. The top r genes were used in this process, where $r = 2,3,4$ and 5. The results are shown in Tables S3.1, S3.2 and S3.3. It can be observed that DRAGON is giving competitive results on all the three datasets using InfoGain.

Table S3.1: Clustering accuracy (%) on acute leukemia dataset.

Methods	Dim 2	Dim 3	Dim 4	Dim 5
SLINK	65.3	63.9	66.7	81.9
CLINK	75.0	70.8	81.9	81.9
ALINK	65.3	73.6	84.7	81.9
Wa-LINK	93.1	93.1	81.9	81.9
Wt-LINK	90.3	88.9	81.9	72.2
MLINK	90.3	91.7	81.9	81.9
SLINK (Div)	65.3	66.7	66.7	81.9
CLINK (Div)	77.8	84.7	80.6	80.6
ALINK (Div)	66.7	66.7	66.7	66.7
Dunn's original (Div)	66.7	77.8	80.6	75.0
Dunn's variant (Div)	65.3	72.2	70.8	70.8
Macnaughton-Smith et al. (Div)	90.3	84.7	81.9	81.9
Principal Direction (Div)	91.7	93.1	88.9	88.9
DRAGON	94.4	91.7	97.1	94.0

Table S3.2: Clustering accuracy (%) on MLL dataset.

Methods	Dim 2	Dim 3	Dim 4	Dim 5
SLINK	40.3	40.3	43.1	43.1
CLINK	45.8	50.0	54.2	73.6
ALINK	50.0	50.0	50.0	43.1
Wa-LINK	62.5	62.5	62.5	61.1
Wt-LINK	45.8	50.0	43.1	43.1
MLINK	45.8	50.0	43.1	43.1
SLINK (Div)	41.7	41.7	43.1	43.1
CLINK (Div)	54.2	45.8	56.9	63.8
ALINK (Div)	41.7	41.7	43.1	43.1
Dunn's original (Div)	44.4	44.4	45.8	44.4
Dunn's variant (Div)	41.7	41.7	43.1	43.1
Macnaughton-Smith et al. (Div)	54.2	48.6	50.0	52.8
Principal Direction (Div)	62.5	62.5	62.5	59.7
DRAGON	68.8	62.8	62.0	61.0

Table S3.3: Clustering accuracy (%) on mutation dataset.

Methods	Dim 2	Dim 3	Dim 4	Dim 5
SLINK	77.9	77.9	82.0	77.9
CLINK	77.3	82.0	70.6	77.7
ALINK	77.3	77.3	75.5	75.5
Wa-LINK	77.3	77.7	51.6	51.0
Wt-LINK	77.3	82.0	70.6	77.3
MLINK	54.6	54.6	77.9	75.5
SLINK (Div)	54.5	54.5	54.5	54.5
CLINK (Div)	54.5	54.5	54.5	54.5
ALINK (Div)	77.9	82.6	82.6	81.1
Dunn's original (Div)	77.9	83.0	82.6	76.3
Dunn's variant (Div)	54.5	54.5	54.5	54.5
Macnaughton-Smith et al. (Div)	77.9	83.0	83.0	76.7
Principal Direction (Div)	54.5	54.5	51.0	51.6
DRAGON	77.9	82.2	82.2	82.2
